# War Remedies

**Published:** 1887-02

**Authors:** 


### WAR REMEDIES.

It would be a matter of interest, as well as of practical usefulness, to gather up and publish the many remedies used during the late war by the Confederate physicians, when by reason of the blockade the supply of drugs was cut off, and it became necessary to use such indiginous herbs, roots, etc., as the wits and independent resources of the doctor suggested. We well remember that poplar, dogwood, and willow barks were used in intermittent fevers as substitutes for quinine, and with fair success. The root of verbascum or mullein was used also for chills, and as a tonic and alterative in bronchial affections—a strong tea being made of the fresh root.

On one occasion, having ordered our children to pull up some dog fennel, the inside of their hands were badly blistered. This suggested dog fennel as an epaspastic, and we afterwards used it with decided effect for that purpose. The fresh leaves of the plant, bruised, moistened and bound to the part will in a short time blister the skin. If kept on long the irritation will be too active, and the inflamed surface will require some days to recover.

We would be pleased to have reported by any of the surviving surgeons of that eventful time any remedies which they may have found useful in the emergencies of practice as substitutes for the ordinary drugs of the shops.

